# The moderating influence of perceived government information transparency on COVID-19 pandemic information adoption on social media systems

**DOI:** 10.3389/fpsyg.2023.1172094

**Published:** 2023-06-19

**Authors:** Isaac Kofi Mensah, Muhammad Khalil Khan, Juan Liang, Nan Zhu, Li-Wei Lin, Deborah Simon Mwakapesa

**Affiliations:** ^1^Business Administration, Fujian Jiangxia University, New University Campus, Fuzhou, Fujian, China; ^2^Department of Journalism and Communication, School of Media and Law, NingboTech University, Ningbo, China; ^3^School of Civil and Surveying Engineering, Jiangxi University of Science and Technology, Ganzhou, Jiangxi, China

**Keywords:** COVID-19 pandemic, public health, information adoption model (IAM), social media systems, perceived government information transparency, Ghana Africa

## Abstract

**Introduction:**

Social media systems are instrumental in the dissemination of timely COVID-19 pandemic information to the general population and contribute to the fight against the pandemic and waves of disinformation during the COVID-19 pandemic. This study uses the information adoption model (IAM) as the theoretical framework to examine the moderating influence of perceived government information transparency on the adoption of COVID-19 pandemic information on social media systems from the Ghanaian perspective. Government information transparency regarding the pandemic is crucial since any lack of transparency can negatively affect the global response to the pandemic by destroying trust (in government and public health authorities/institutions), intensifying fears, and causing destructive behaviors.

**Methods:**

It applies a convenient sampling technique to collect the responses from 516 participants by using self-administrated questionnaires. The data analysis was computed and analyzed with SPSS-22. The following statistical tests were conducted to test the hypotheses: descriptive statistics, scale reliability test, Pearson bivariate correlation, multiple linear regressions, hierarchical regression, and slope analysis.

**Results:**

The results indicate that information quality, information credibility, and information usefulness are significant drivers of COVID-19 pandemic information adoption on social media systems. Furthermore, the perceived government information transparency positively moderates the influence of information quality, information credibility, and information usefulness on the adoption of COVID-19 pandemic information on social media systems.

**Conclusion:**

The theoretical and managerial implications of these findings suggest the utilization of social media systems as an effective tool to support the continued fight against the current COVID-19 pandemic and its future role in national and global public health emergencies.

## 1. Introduction

The unexpected emergence of the Coronavirus Disease (COVID-19) pandemic which was first detected in the Chinese City of Wuhan in late December 2019 has overwhelmed the world with its stringent impact on people’s social, physiological, psychological, and economic well-being (Clemente-Suárez et al., 2020; Murshed, 2022; Panneer et al., 2022). The effect of the COVID-19 pandemic on both the social and economic well-being of the people pushes countries to develop and implement diverse social, community, economic, and public health prevention and control mechanisms to reduce the high mortality rates and stop the likelihood of collapse in their country’s social, health and economic systems (Clemente-Suárez et al., 2020; Assefa et al., 2022). Social media systems became one of the readily available social systems to governments and authorities in charge of COVID-19 management to adequately disseminate information (preventive and control measures) about the pandemic to its citizens and the general population (Xie et al., 2022; Nahidh et al., 2023). It plays an instrumental role in reducing the rate of infection and to a large extent the number of deaths since people who were well informed about the preventive and control measures of COVID-19 took subsequent steps to protect themselves from the pandemic (Mohammed et al., 2023; Song et al., 2023).

Social media as a mass media technology is the major source of information due to ease and inexpensive access to the internet with the large user base(Hayes, 2022; Papademetriou et al., 2022). This makes it one of the best and most effective mechanisms for information dissemination (González-Padilla and Tortolero-Blanco, 2020; Bayer et al., 2022). It has been useful in public health communications, especially during the periods of the current COVID-19 pandemic due to its flexibility and pervasiveness to encourage people to stick to preventive and control measures to fight the spread of the pandemic (González-Padilla and Tortolero-Blanco, 2020; Hussain, 2020; Hamilton et al., 2022). Social media consequently encourage positive health attitudes and behaviors as well as adherence to pragmatic control and preventive COVID-19 protocols (Hussain, 2020; Papademetriou et al., 2022). Hence, social media systems such as WhatsApp, Facebook, Twitter, Instagram, YouTube, and Snapchat have contributed positively in the combat of the pandemic through the advancement of good policies/strategies to assist people to confront social and physical distancing by reducing the stigma, discrimination, prejudices, and inequalities associated with the COVID-19 pandemic (Hussain, 2020; Marciano et al., 2022). It has been highlighted that social media characteristics that were relevant in the pandemic have to do with the faster dissemination of COVID-19 preventive and control measures at different levels (district, regional, national, and international levels) (González-Padilla and Tortolero-Blanco, 2020; Sharma and Kapoor, 2022).

Furthermore, social media systems have been used by new agencies, organizations, and society to share information and disinformation about the pandemic (Ravichandran and Keikhosrokiani, 2023). Misinformation entails information that is incorrect but not intended to deceive or cause harm while disinformation is false information that is intentionally created to harm a person, social group, organization, or country and it is often motivated by financial or political purposes (Gisondi et al., 2022). Those forms of information falsification are prevalent across all social media systems and they jointly account to undermine trust in government prevention and control interventions, public health responses, expert guidance, and scientific facts/data concerning the COVID-19 pandemic (Gottlieb and Dyer, 2020; Gisondi et al., 2022). Also, misinformation is deceptive information that is considered misleading/incorrect and drive the spreading of the COVID-19 virus (Joseph et al., 2022). Terms such as fake news, rumors, conspiracy theories, false information misleading information, are often connected to the misinformation paradigm (Sanaullah et al., 2022). People’s behavior (unhealthy health behaviors and unsound practices) which is based on the accuracy or inaccuracy of the information are crucial to the global management health response to the pandemic (Sanaullah et al., 2022; Ravichandran and Keikhosrokiani, 2023). This is buttressed by reports indicating that COVID-19 misinformation presents a huge risk to public health and public action to fight the unprecedented nature of the COVID-19 pandemic (Barua et al., 2020; Bridgman et al., 2020). The rapid spread of COVID-19 misinformation on social media effect government COVID-19 pandemic management efforts and thus has major consequences on public health responses. It increases fear among the people leading to severe damage to the physical and mental well-being of individuals (Sanaullah et al., 2022; Wang et al., 2022). Consequently, there have been concerted efforts globally to combat the spread of COVID-19 pandemic misinformation on social media systems (Sanaullah et al., 2022).

Ghana’s government widely uses social media systems to communicate COVID-19-related information to the general public and social media systems remained a vital source of information for the Ghanaian public about COVID-19 pandemic management information(Adu-Gyamfi and Asante, 2022; Britwum et al., 2023). Eyifah (2021) noted that 59.5% of Ghanaians use social media systems, particularly Facebook and WhatsApp as the main source to obtain information related to the COVID-19 pandemic management. The prevalence of social media presents some challenges and opportunities for information transparency and engagement in transparent communication is the key to building trust (DiStaso and Bortree, 2012). COVID-19 pandemic and increasing demand for transparency has intensified the importance of sustainability in transparent communications on social media systems (Mann et al., 2021). Information transparency is characterized by elements such as truthfulness, substantial/useful, objective, balanced, and accountability (DiStaso and Bortree, 2012). Developing a strong digital information ethics management, improving digital ethics for virtual communication skills, and expanding digital ethics in information quality are fundamental to achieving social media information transparency (Huda et al., 2022). Reports indicate that though governments were effective in the use of social media to communicate and disseminate COVID-19 pandemic information, it, however, lacked transparency (El Baradei et al., 2021). Achieving greater transparency strengthens public confidence and is considered the best form of defense against misunderstanding, misuse, and premeditated misinformation about the COVID-19 pandemic (Barton et al., 2020). Thus, information transparency becomes the catalyst weapon to reduce the menace of COVID-19 misinformation and disinformation. Transparency in the presentation of government COVID-19 information could contribute to creating social susceptibility and solidarity (Ölcer et al., 2020). Therefore, government information transparency regarding the pandemic is crucial since any lack of transparency can affect the global response to the pandemic by destroying trust (in government and public health authorities/institutions), intensifying fears, and causing destructive behaviors (Mansoor, 2021; Oldeweme et al., 2021; OECD, 2023).

Thus, this study investigates the moderated influence of perceived government information transparency on the adoption of COVID-19 pandemic information on social media systems from the Ghanaian perspective. This was done by integrating government information transparency into the Information Adoption Model (IAM). The information adoption model though has been used to understand information adoption, management, and dissemination during the COVID-19 pandemic (Han et al., 2021; Mensah et al., 2022), these studies, however, fail to consider how government COVID-19 information transparency on social media systems can influence the major constructs in the IAM such as information quality, information credibility and information usefulness on the adoption of COVID-19 pandemic information on social media systems. This gap is vital since the nature of COVID-19 information shared on social media systems has the power to drive people’s behavioral response and may to some extent change the effectiveness of the counter mechanisms deployed by governments to fight the pandemic (Cinelli et al., 2020). It has been further emphasized that the lack of transparency on the part of government information dissemination relating to the COVID-19 pandemic has the potential to decrease the success of dealing with the COVID-19 pandemic (Pramiyanti et al., 2020). It has additionally been advocated that transparency in COVID-19 communication is important since transparency breeds trust in health authorities as well as the government and crucially it can prevent the spread of misinformation and disinformation (i.e., conspiracy theories) about the pandemic (Petersen et al., 2021). Therefore, this study investigated: To what extent does government COVID-19 information transparency on social media systems moderate the influence of information quality, information credibility, and information usefulness on the citizens’ adoption of COVID-19 pandemic information on social media systems?

The paper is structured as follows: background of the study, research theoretical framework, model and hypothesis development, methods, results, discussion and practical implications, theoretical implications, conclusions and limitations, and future works.

### 1.1. Background of the study

The COVID-19 pandemic has gravely impacted the social, psychological, and economic life of Ghanaian people and adversely affected the country’s economic growth (Amewu et al., 2020). The World Health Organization report indicates that a total of 1,771,112 (5.39% of the country’s total population, i.e., 32,843,530 million) confirmed COVID-19 cases with 1,462 (0.0044% of the country’s total population) deaths were reported in Ghana from 3 January 2020 to 3 February 2023 (WHO, 2023). Additionally, the Ghana government has administered successfully a total of 22,384,226 (68.15% of the country’s total population) doses of vaccines in Ghana as of 14 January 2023 (WHO, 2023). Since the inception of the pandemic in Ghana, there have been 14 Presidential Addresses from the seating President of the Republic of Ghana on the COVID-19 pandemic (detailing the public health and social measures) in the country to the general population (Gyamfi and Amankwah, 2021).

Similarly, social media systems in Ghana such as Facebook, WhatsApp, Instagram, Twitter, LinkedIn, and YouTube are crucial to the dissemination of Government COVID-19-related information to the public. Facebook and Whatsapp have been confirmed to be the most utilized social media system by the government and its agencies in communicating COVID-19 pandemic-related information (Eyifah, 2021). Furthermore, research has reported that social media is vital as a tool for disseminating risks, crises, and preventive mechanisms of the pandemic among Ghana citizens with data showing that the majority of citizens (91%) had indicated in a survey that they had read COVID-19 pandemic related information on social media systems (Eyifah, 2021). The same study revealed that the majority of respondents (83.2%) had indicated that they had read COVID-19 pandemic-related information on government social media pages and the government agency‘s social media pages from which COVID-19 information was read mostly originated from the Ministry of Health (54%) and Ministry of Information (50.5%) (Eyifah, 2021).

## 2. Research theoretical framework

### 2.1. Information adoption model

The information adoption model (IAM) developed by Sussman and Siegal (2003) is based on the theories of adoption and informational influence to explain the drivers of people’s adoption of information in the organizational context. The IAM was developed based on the Theory of Reasoned Action (TA; Hair et al., 2010) and the technology acceptance model (TAM; Davis, 1989) which fundamentally opinionated that beliefs (such as perceived usefulness and perceived ease of use) are core factors influencing the individual behavior to adopt information technological systems. Concerning information adoption, the belief of the perceived usefulness of information is a critical component of individual beliefs that influences the adoption of information.

**Figure 1:**
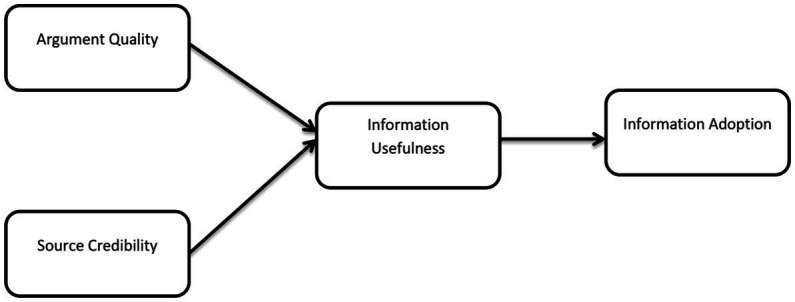
Information adoption model (IAM; Sanaullah et al., 2022).

Furthermore, another core aspect of the IAM is the elaboration likelihood model (ELM) which examines how people are influenced to utilize information shared on computer-mediated communication systems (Sussman and Siegal, 2003). The elaboration likelihood model (ELM) suggests that a delivered message can impact an individual user’s attitudes and behaviors in two dimensions known as centrally and peripherally (Sussman and Siegal, 2003). The central dimension is concerned with the nature of the arguments presented in the message and the peripheral aspect looks at themes that are not directly connected to the subject matter presented in the message (Sussman and Siegal, 2003). The Information Adoption Model (IAM) depicted in Figure 1 has two core components such as argument quality (also known as information quality) which is considered the central influence and source credibility which is referred to as the peripheral influence (Sussman and Siegal, 2003). These two core components are considered to be the key drivers of information usefulness which consequently influences information adoption.

## 3. Research model and hypothesis development

### 3.1. Research model

**Figure 2:**
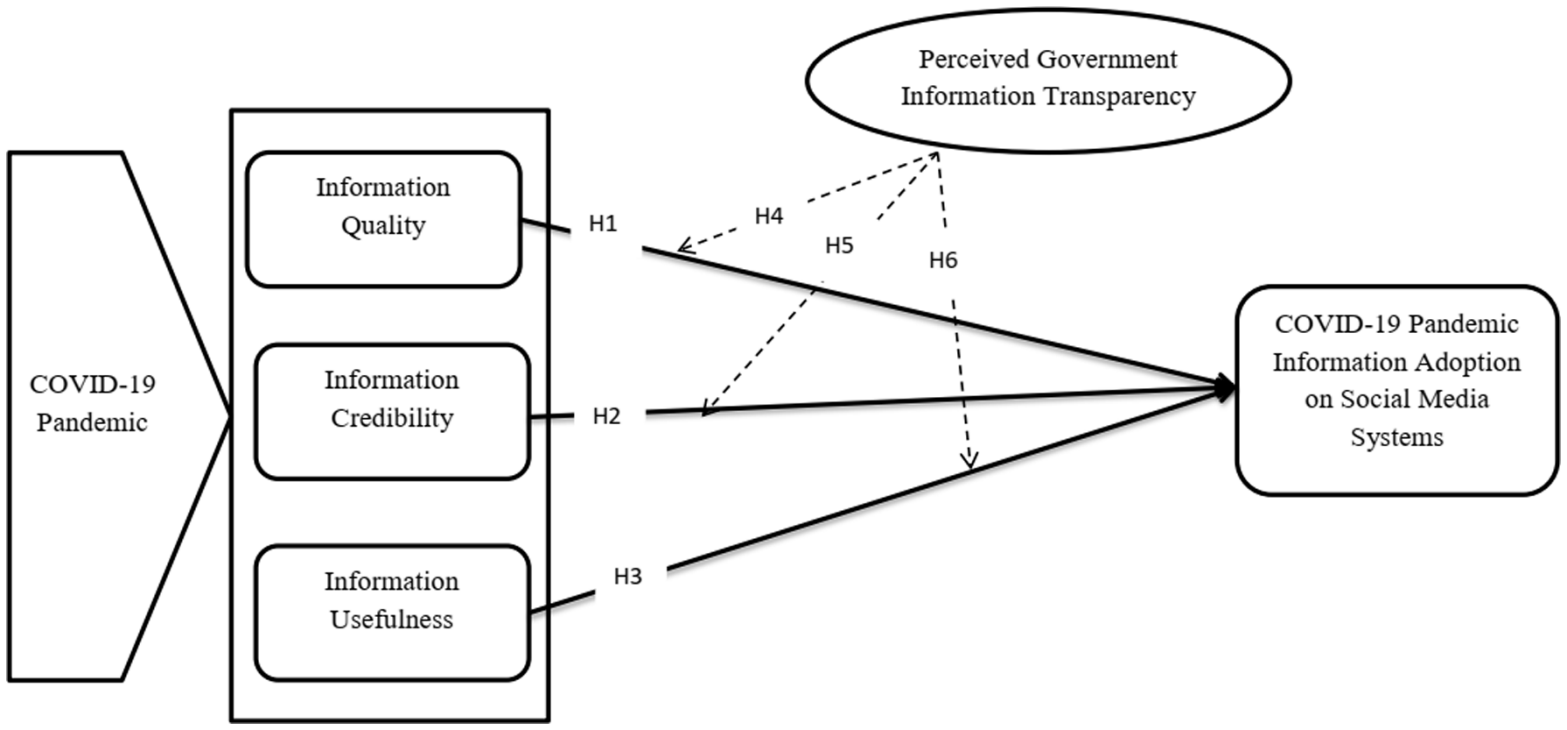
Research model.

The study research model is based on the research hypothesis developed in the following section (Figure 2). Information quality, information credibility, and information usefulness are anticipated to influence COVID-19 pandemic information adoption on social media systems. Additionally, perceived government information transparency is projected to moderate the influence of information quality, information credibility, and information usefulness on the COVID-19 pandemic information adoption on social media systems.

### 3.2. Hypothesis development

#### 3.2.1. Information quality

Information quality which is also described as argument quality is considered as the persuasive strength of information/arguments inherent in the information provided and it is seen as the values of output created by a system as interpreted by the receiver of the information (Sussman and Siegal, 2003; Beatrix, 2022). Information quality is often evaluated by individual users based on the accuracy, formation, relevance, currency, timeliness, understandability, dynamism, completeness, and content of the information that the system provides (Dwiputra et al., 2021; Simanjuntak et al., 2022). Information quality relating to COVID-19 has to do with the fit for purpose and use of the pandemic information provided on social media systems. Social media provided swift medium and access which saw the publication of high volumes of COVID-19 pandemic-related information online which sometimes questioned the quality of such information provided on social media systems. Consequently, COVID-19 information that is shared on social media that demonstrates these information quality characteristics such as accuracy, relevance, timeliness, completeness, and understandability, etc. would be instrumental in driving the individual adoption behavior of COVID-19 pandemic information on social media. That is if individual users can visualize and internalize the information quality of COVID-19 information in terms of the information quality standards (accuracy, relevance, timeliness, completeness, and understandability) delivered on social media systems they will be attracted to utilize COVID-19 information shared on social media systems. The impact of information quality on the adoption of COVID-19-related information has been validated by previous works (Mensah et al., 2022). Accordingly, H1 was advanced.

Hypothesis 1: Information quality positively influences the COVID-19 pandemic information adoption on social media systems.

#### 3.2.2. Information credibility

Information credibility is considered as a receiver-dependent evaluation that includes both objective understanding/perceptions of the message credibility and subjective judgment of the medium credibility (Li and Suh, 2015; Kang and Namkung, 2019). It is the degree to which individuals perceive the information provided to be believable and is a strong determinant of users’ reaction to their information consumption behavior in terms of its recommendation and readiness to share the information received (Li and Suh, 2015; Keshavarz, 2021). It is also the extent to which the information received is considered to be believable, competent, and trustworthy by the receiver of the information (Cooley and Parks-Yancy, 2019). Social media is a vital source of information during the COVID-19 pandemic however the credibility of social media content is still a major concern and thus determining the credibility of information on social media is a huge task/challenge (Aladhadh et al., 2019; Benis et al., 2021). It has been highlighted that the credibility of information can be measured via knowledge concerning the source with identifiable information sources being more credible than anonymous ones (Aladhadh et al., 2019). Furthermore, it has been confirmed that the majority of social media posts regarding the COVID-19 pandemic were not highly reliable and trustworthy. However, social media posts shared by authoritative personalities like doctors, medical practitioners, etc. were considered reliable and were more trusted by users (Tayal and Bharathi, 2021). It thus follows that COVID-19 information shared on social media that demonstrate high levels of credibility will encourage more users to adopt COVID-19 pandemic information on social media. Studies have confirmed that information credibility is significantly associated with information adoption on social media (Khan et al., 2022; Mensah et al., 2022). Consequently, H2 was put forward.

Hypothesis 2: Information credibility positively influences the COVID-19 pandemic information adoption on social media systems.

#### 3.2.3. Information usefulness

Perceived usefulness is considered the extent to which users understand that the use of new information technology systems will contribute to improving or enhancing his/her performance (Davis, 1989; Marangunić and Granić, 2015). It is further described as how users consider the information received to be valuable and it is a major driver of information adoption (Sussman and Siegal, 2003; Luo et al., 2018). COVID-19 pandemic information usefulness on social media is the perception of social media users that the COVID-19 pandemic-related information disseminated on social media will be valuable in contributing to enhancing knowledge of the virus and thus reduce the chance of any infections and impact of the pandemic on his or her life. Consequently, if users of social media consider the information shared concerning the COVID-19 pandemic to be useful in improving their knowledge of the pandemic as well as the protective protocols (social and physical distancing, testing, vaccination, masks wearing, going to high-risk areas and hygiene steps, etc.). They will happily follow and adopt such information on social media to reduce the adverse impact of the pandemic on their personal and family lives. Hence, research has shown that information usefulness is significantly associated with the adoption of information (Han et al., 2021; Shang et al., 2021; Mensah et al., 2022). Thus, H3 was advanced.

Hypothesis 3: Information usefulness positively influences the COVID-19 pandemic information adoption on social media systems.

#### 3.2.4. Perceived government information transparency

Governments are morally and legally mandated to provide relevant information to the general public in times of public disasters and global emergencies like the deadly COVID-19 pandemic. The provisions of information in times of national and global emergencies to the public are expected to be open, transparent, and devoid of any political manipulation and distortions. That is the provisions of public information and communication for public consumption should be factual, transparent, and separate from political communication (OECD, 2023). Openness and transparency in the provision of public information are essential tenets of good governance and effectively control and manage the pandemic (Jannah and Sipahutar, 2022). Therefore, transparency in government information provision drives active public participation in decision-making, community compliance and increases the public trust in government during the COVID-19 pandemic (Pramiyanti et al., 2020; Jannah and Sipahutar, 2022). It has been further elaborated that the success of the government’s COVID-19 pandemic control mechanism depends solely on the public trust and acceptance of COVID-19 measures which can be forged on perceived transparency of COVID-19 information (Enria et al., 2021). It has also been confirmed that countries encountered challenges in the manner in which they presented and disseminated COVID-19 pandemic information and these issues concerned the need for simplicity, use of open data, transparency, and trust (WHO, 2022). Aside from the traditional media, governments actively use social media systems to share and disseminate information on the COVID-19 pandemic to the general public to drive people’s disclosure of information, demonstrate the government’s commitment, increase citizens’ preparedness and awareness generation (Li et al., 2020; Padeiro et al., 2021). Consequently, the nature of government COVID-19 information transparency shared on social media has the potential to moderate the influence of information quality, information credibility, and information usefulness on the adoption of COVID-19 information on social media. Accordingly, H4, H5 and H6 were proposed.

Hypothesis 4: Perceived government information transparency positively moderates the influence of information quality on the COVID-19 pandemic information adoption on social media systems.

Hypothesis 5: Perceived government information transparency positively moderates the influence of information credibility on the COVID-19 pandemic information adoption on social media systems.

Hypothesis 6: Perceived government information transparency positively moderates the influence of information usefulness on the COVID-19 pandemic information adoption on social media systems.

## 4. Methods

### 4.1. Sampling and procedures

A self-administered research questionnaire was designed to gather the data to test the research hypothesis and model. The questionnaire items were adapted from previous literature but they were restructured to conform to the specific context as well as to achieve the objectives of this paper. The questionnaire items were taken from the following sources as outlined: information quality (Mensah et al., 2022), information credibility (Elwalda et al., 2021), information usefulness (Mensah et al., 2022), perceived government information transparency (was self-developed), and COVID-19 pandemic information adoption (Han et al., 2021).

A piloting and pre-testing of the questionnaire were carried out with 20 people to test if there was difficulty in understanding any of the statements in the questionnaire. Piloting and pretesting ensure that questions are articulated clearly and that the response options are relevant, comprehensive, and mutually exclusive (researchers and respondents understand the survey in the same manner; Chan and Holosko, 2020; Geisen and Murphy, 2020). The feedback from the pretesting and piloting cause some of the question items/statements to be rechecked and restructured for simplicity and clarity of language. It further ensured that any potential form of ambiguity that respondents may have in comprehending the contents of the questionnaire was reduced and removed. The results from the piloting and pre-testing were however not included in the final data analysis due to their small nature.

This study used a convenient sampling technique which is effective, efficient, and inexpensive to reach the respondents for data collection. Convenient sampling empowers researchers to acquire respondents that are closer and more convenient to the researcher and thus provides readily available samples at low cost (Burke et al., 2020; Andrade, 2021). The questionnaire was hosted online (Qualtrics online survey software) and the link created was distributed through WhatsApp social media system to a cross-section of Ghanaian citizens who are from three university communities and its environs namely the Ghana Institute of Management and Public Administration (GIMPA), University of Professional Studies Accra (UPSA) and the University of Ghana. WhatsApp social media was used since it is among the most popular social media messaging app used in Ghana and thus empowers the researchers to reach the respondents faster for data collection. Friends and known contacts in those universities were also used to share the link to their immediate WhatsApp contacts. The data collection period lasted for 3 months (from September to November 2022) of which a total of 516 valid responses were acquired. The questionnaire hosted online was designed in a manner that no respondents can submit a questionnaire without completing all the sections of the questionnaire and thus the issues of incomplete as well as invalid responses were eliminated.

### 4.2. Measures

#### 4.2.1. Information quality

Information quality was measured through a three-item scale adopted from previous research (Mensah et al., 2022): (a) comprehensiveness and objectivity (that is, COVID-19 pandemic information on social media is comprehensive and objective); (b) timely and updated (COVID-19 pandemic information on social media is timely and updated); (c) accurate and relevant (COVID-19 pandemic information on social media is accurate and relevant). A five-point Likert scale (1 = strongly disagree, 5 = strongly agree) was used. Those three items were combined to form an information quality scale (*M* = 4.27, SD = 0.710, α = 0.94).

#### 4.2.2. Information creditability

Information creditability was measured on a three-item scale adopted from a previous research study (Elwalda et al., 2021): (a) believability (that is, COVID-19 pandemic information on social media is believable); (b) factuality (COVID-19 pandemic information on social media is factual); (c) trustworthiness (COVID-19 pandemic information on social media is trustworthy). A five-point Likert scale (1 = strongly disagree, 5 = strongly agree) was used. Those three items were combined to form an information credibility scale (*M* = 4.20, SD = 0.610, α = 0.83).

#### 4.2.3. Information usefulness

Information usefulness was measured on a three-item scale adopted from a previous research study (Elwalda et al., 2021; Mensah et al., 2022): (a) usefulness (that is, COVID-19 pandemic information on social media is useful); (b) beneficial (COVID-19 pandemic information on social media is beneficial to protect me from virus); (c) relevant (COVID-19 pandemic information on social media provides all the relevant knowledge I need to know about pandemic). A five-point Likert scale (1 = strongly disagree, 5 = strongly agree) was used. Those three items were combined to form an information usefulness scale (*M* = 4.32, SD = 0.692, α = 0.88).

#### 4.2.4. Perceived government information transparency

The perceived government information transparency scale was self-developed based on literature reviews and comprises three items: (a) openness (I think the government is open regarding COVID-19 pandemic information shared on social media); (b) communicative (I think the government COVID-19 pandemic information shared on social media is not secretive but rather communicative); (c) availability [I think the government provides all available information on social media concerning COVID-19 pandemic management (i.e., infected cases and deaths)]. A five-point Likert scale (1 = strongly disagree, 5 = strongly agree) was used. Those three items were combined to form a perceived government information transparency scale (*M* = 4.41, SD = 0.689, α = 0.96).

#### 4.2.5. Information adoption on social media systems

The COVID-19 pandemic information adoption scale was adopted from a previous research study (Han et al., 2021) that comprises three items: (a) usage (I plan to make usage of COVID-19 pandemic information on social media); (b) adoption (I will frequently adopt COVID-19 pandemic information on social media); (c) recommendation (I will recommend other to use COVID-19 pandemic information on social media). A five-point Likert scale (1 = strongly disagree, 5 = strongly agree) was used. Those three items were combined to form the COVID-19 pandemic information adoption scale (*M* = 4.13, SD = 0.777, α = 0.94).

### 4.3. Data analysis

We used 516 valid responses for the data analysis that are computed and analyzed with SPSS-22. The following statistical tests were conducted to test the hypotheses: descriptive statistics, scale reliability test, Pearson bivariate correlation, multiple linear regressions, hierarchical regression, and slope analysis. Since the study relied on self-reporting measures, a common method variance was tested. Therefore, we applied Harman’s single-factor analysis to examine the common method bias (CMB) in this study (Podsakoff et al., 2003). Results indicate that a single factor has extracted 27.045% of the total variance which is far less than 50% of the total variance. In addition, we have not found a high bivariate correlation between variables (see Table 1). So, there is no CMB found in this study.

**Table 1:** Descriptive statistics of main variables.

	Mean	SD	1	2	3	4	5
1. Information quality	4.270	0.710	1				
2. Information credibility	4.205	0.610	0.350***	1			
3. Information utility	4.323	0.692	0.240***	0.385***	1		
4. Perceived government information transparency	4.405	0.669	0.154***	0.177***	0.135**	1	
5. Information adoption on social media system	4.134	0.777	0.150**	0.166***	0.156***	0.271***	1

**p < 0.01, ***p < 0.001.

### 4.4. Demographics

A total of 516 respondents (273 females [52.9%] and 243 males [47.1%]) participated in this study. The majority of the respondents hold bachelor’s (*N* = 197, 38.2%) and master’s degrees (*N* = 157, 30.4%). Whereas 16.7% (*N* = 86) hold Ph.D. degrees and other 14.7% (*N* = 76) hold technical education. Participants professionally belong to university teaching staff (*N* = 82, 15.9%), non-teaching staff (*N* = 158, 30.6%), students (*N* = 183, 35.5%), and other technical staff (*N* = 93, 18.0%).

## 5. Results

### 5.1. Descriptive statistics

Descriptive statistics indicate the means, standard deviations, and correlations of all the main variables (Table 1). All main variables, i.e., information quality (IQ), information credibility (IC), information utility (IU), perceived government information transparency (PGIT), and COVID-19 information adoption on social media system (IAoSMS) are positively correlated with each other and their means and standard deviation are in the expected direction of this study.

### 5.2. Reliability analysis

Based on previous research (Hair et al., 2010; Hair, 2011), we applied exploratory factor analysis by using the principle component analysis to examine the internal reliability of constructs by extracting similar factor loadings. Additionally, Cronbach’s alpha, composite reliability test, and average variance extracted (AVE) were also used to further demonstrate the reliability and validity of the constructs in our study. Researchers (Hair et al., 2010; Hair, 2011) recommended factor loadings of a minimum value of 0.600, composite reliability and Cronbach alpha threshold values of 0.700, and AVE a value of not less than 0.500v. As indicated in Table 2, the entire requirement value thresholds have been met and thus demonstrate the construct reliability of the measures.

**Table 2:** Reliability analysis of the questionnaire.

Construct	Items	Factor loading	Cronbach alpha	Composite reliability	Average variance extracted
Information quality (IQ)	IQ1: COVID-19 pandemic information on social media is comprehensive and objective	0.945	0.945	0.953	0.870
	IQ2: COVID-19 pandemic information on social media is timely and updated	0.952			
	IQ3: COVID-19 pandemic information on social media is accurate and relevant	0.901			
Information credibility (IC)	IC1: COVID-19 pandemic information on social media is believable	0.904	0.831	0.873	0.699
	IC2: COVID-19 pandemic information on social is media factual	0.891			
	IC3: COVID-19 pandemic information on social media is trustworthy	0.697			
Information usefulness (IU)	IU1: COVID-19 pandemic information on social media is useful	0.956	0.878	0.915	0.784
	IU2: COVID-19 pandemic information on social media is beneficial to protect me from the virus	0.731			
	IU3: COVID-19 pandemic information on social media provides all the relevant knowledge I need to know about the pandemic	0.951			
Perceived government information transparency (PGIP)	PGIP1: I think the government is open regarding the COVID-19 pandemic information shared on social media	0.936	0.962	0.970	0.914
	PGIP2: I think the government COVID-19 pandemic information shared on social media is not secretive but rather communicative	0.971			
	PGIP3: I think the government provides all available information on social media concerning COVID-19 pandemic management (i.e., infected cases and deaths)	0.961			
Information adoption on social media systems (IAoSMS)	IAoSMS1: I plan to make use of COVID-19 pandemic information on social media	0.937	0.936	0.953	0.871
	IAoSMS2: I will frequently adopt COVID-19 pandemic information on social media	0.909			
	IAoSMS3: I will recommend to others to use COVID-19 pandemic information on social media	0.953			

### 5.3. Regression analysis

To test H1, H2, and H3, we used multiple linear regression analysis. We predict that information quality, information credibility, and information utility are positively connected to the COVID-19 pandemic information adoption on social media systems. Results indicate that information quality positively influenced the COVID-19 pandemic information adoption (*β* = 0.102, *p* < 0.05) on social media systems (Table 3). Likewise, the information credibility also positively influenced the COVID-19 pandemic information adoption (*β* = 0.123, *p* < 0.05) on social media systems. The information usefulness also positively influences the COVID-19 pandemic information adoption on social media systems (*β* = 0.109, *p* < 0.05). Hence the model significantly supported the H1, H2, and H3 (*R*^2^ = 0 0.045, *p* < 0.001) respectively (Table 3).

**Table 3:** Multiple regression analysis.

Hypotheses	Variables	Information adoption on social media systems	Decision
	**Main effects**		
H1	Information quality (IQ)	0.102*	H1 supported
H2	Information creditability (IC)	0.123*	H2 supported
H3	Information utility (IU)	0.109*	H3 supported
	*R* ^2^	0.045	
	Adjusted *R*^2^	0.039	
	*F*	8.059***	

***p < 0.001, *p < 0.05.

### 5.4. Moderation hypotheses analysis

We used the most commonly used moderation approach (Shalley et al., 2009; Zwikael et al., 2014) to test the moderation effects of perceived government information transparency (PGIT) on the relationship between information quality (IQ) and COVID-19 pandemic information adoption on social media systems (IAoSMS); between information credibility (IC) and IAoSMS; and between information usefulness (IU) and IAoSMS. We applied Stone-Romero and Anderson (1994) moderated hierarchical regression model to validate the moderation hypotheses, i.e., H4, H5, and H6. First, we centralized the independent variables (IQ, IC, and IU) and moderator variable (PGIT). Then, we entered the product term of the independent and moderation variable (IQ × PGIT; IC × PGIT; IU × PGIT) into the hierarchical regression model. The results indicate that PGIT positively moderates (*β* = 0.144, *p* < 0.05) the influence of information quality on the COVID-19 pandemic information adoption on social media systems. Likewise, PGIT positively moderates (*β* = 0.049, *p* < 0.001) the influence of information credibility on the COVID-19 pandemic information adoption on social media systems. However, the PGIT moderation effects on the relationship between information usefulness and the COVID-19 pandemic information adoption on social media systems are relatively low (*β* = 0.104, *p* < 0.10). We used demographic variables (age, gender, education, and profession) as the control variable in this analysis. The model significantly supported the H4, H5, and H6 (*R*^2^ = 0.096, *p* < 0.001; Table 4).

**Table 4:** Moderation analysis.

Hypotheses	Variables	Information adoption on social media systems (IAoSMS)	Decision
	**Control variables**		
	Gender	−0.049	
	Age	0.048	
	Education	−0.029	
	Profession	−0.119***	
	**Moderation effects**		
H4	IQ × PGIT	0.144*	H1 supported
H5	IC × PGIT	0.049***	H2 supported
H6	IU × PGIT	0.104^†^	H3 supported
	*R* ^2^	0.096	
	Adjusted *R*^2^	0.091	
	*F*	18.174***	

†*p* < 0.10, *p < 0.5, ***p < 0.001, PGIT, Perceived government information transparency.

**Figure 3:**
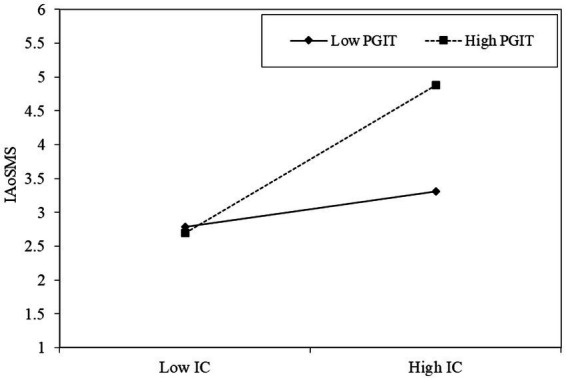
Slope analysis of the moderation effects of perceived government information transparency (PGIT) on the relationship between information quality (IQ) and COVID-19 pandemic information adoption on social media systems (IAoSMS).

To further validate the moderation analysis, we conducted the simple slope analysis which revealed that the relationship between information quality and COVID-19 pandemic information adoption on social media systems is positive (*β* = 0.308, *p* < 0.001) when the perceived government information transparency is high. However, when the perceived government information transparency is low, the relationship between information quality and COVID-19 pandemic information adoption on social media systems is not statistically significant (*β* = 0.114, *p* = ns; Figure 3).

**Figure 4:**
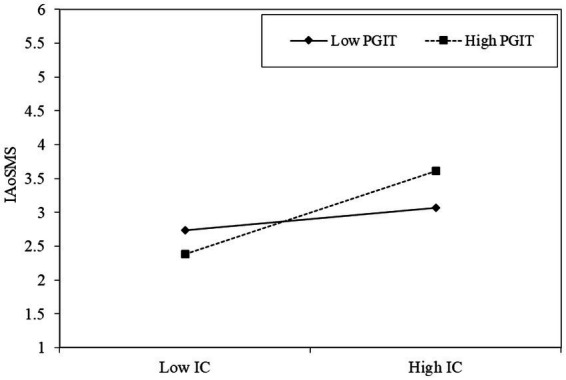
Slope Analysis of the moderation effects of perceived government information transparency (PGIT) on the relationship between information credibility (IC) and COVID-19 pandemic information adoption on social media systems (IAoSMS).

Likewise, the relationship between information credibility and COVID-19 pandemic information adoption on social media systems is positive (*β* = 0.092, *p* < 0.001) when the perceived government information transparency is high and the relationship between information credibility and COVID-19 pandemic information adoption on social media systems is positive but not statistically significant (*β* = 0.013, *p* = ns) when the perceived government information transparency is low (Figure 4).

**Figure 5:**
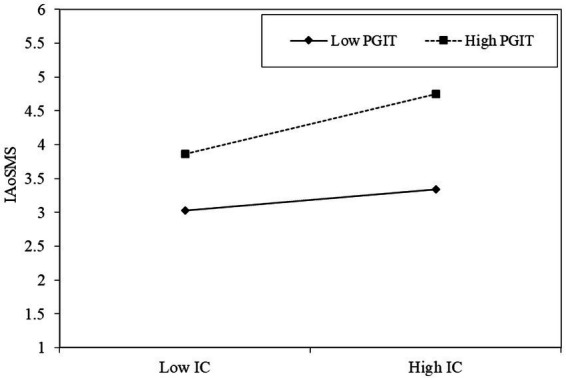
Slope Analysis of the moderation effects of perceived government information transparency (PGIT) on the relationship between information utility (IU) and COVID-19 pandemic information adoption on social media systems (IAoSMS).

However, the relationship between information utility and COVID-19 pandemic information adoption on social media systems is relatively low (*β* = 0.003, *p* < 0.10) when the perceived government information transparency is high and the relationship between information utility and COVID-19 pandemic information adoption on social media systems is not statistically significant (*β* = 0.125, *p* = ns) when the perceived government information transparency is low (Figure 5).

## 6. Discussion

COVID-19 information dissemination and its subsequent adoption are critical to the success of COVID-19 preventive and control measures. This study discusses the importance of information adoption in times of public health emergencies such as the COVID-19 pandemic and examined the moderating influence of perceived government information transparency on the adoption of COVID-19 pandemic information on social media systems. Governments and public sector health institutions are the main bodies in charge of the management and containment of the deadly COVID-19 virus infection among the population especially when it comes to the provision of relevant information about the COVID-19 pandemic for public consumption. Subsequently, the nature of the COVID-19 pandemic information provided by the government tends to influence the success of management strategies instituted to deal with the pandemic as well as people’s behavior regarding the deadly pandemic.

The integration of perceived government information transparency into the Information Adoption Model (IAM) coupled with the data analysis conducted has shown that the core constructs in the IAM such as information quality, information credibility, and information usefulness were significant predictors of the adoption of COVID-19 pandemic information on social media systems. Also, the moderating analysis has demonstrated that perceived government information transparency was significant in moderating the influence of information quality, information credibility, and information usefulness, respectively, on the adoption of COVID-19 pandemic information on social media systems. However, the study found that the influence of perceived government information transparency on the relationship between information usefulness and COVID-19 pandemic information adoption on social media systems is relatively low.

The global nature of the COVID-19 pandemic with its devastating effect on every individual in the world irrespective of the country of origin makes our research findings internationally appealing and thus could be extrapolated though cautiously to other countries. Especially, the amount of COVID-19 pandemic disinformation, distortion, and, misinformation, coupled with the lack of information transparency that heralded the communication and dissemination of COVID-19-related information on social media systems to the public. The findings of this study consequently could be useful to governments, health authorities, organizations, and bodies in charge of COVID-19 response management (preventive and control measures) in other countries to strive to achieve the highest form of information transparency as a strategic response to combat current and future pandemic crisis. The managerial implications of the findings of this paper are therefore applicable both in Ghana and possible implementation elsewhere.

### 6.1. Practical implications

The direct impact of COVID-19 information quality on the adoption of COVID-19 pandemic information on social media systems does demonstrate that the provision of COVID-19 pandemic information by health authorities and government agencies should be done to meet key information quality standards of timeliness, appropriateness, completeness, accuracy, and reliability. COVID-19 information for instance on the prevention and control measures should be communicated promptly and presented completely to demonstrate its accuracy and reliability if it is to be adopted by users on social media systems. To ensure the effective delivery of health information in times of public health crises like the COVID-19 pandemic, it is imperative to provide clear, consistent, and reliable information on the pandemic to help mitigate and control the surge of the outbreak. This finding is in line with other previous studies that have shown that COVID-19 information quality drives the adoption of COVID-19 pandemic information on social media systems (Mensah et al., 2022).

Furthermore, the direct impact of COVID-19 information credibility on the adoption of COVID-19 information on social media systems does illustrate how the credibility of COVID-19 pandemic information can drive people to adopt such information. The absence of relevant professionals to monitor the content of social media information especially the provision of COVID-19 information on social media systems sometimes questions the information credibility of such social media information. COVID-19 information should demonstrate credibility in areas such as subject credibility, source credibility, and content credibility if they are to be adopted. Churning out of credibility, COVID-19 information can ensure the believability of such information by users and thus using their cognitive capacities they can discern genuine information from fake ones. It has been emphasized that given the relevance of assessing the credibility of information, information literacy, and science-based knowledge concerning COVID-19 should be provided (Amit Aharon et al., 2021). Our finding corroborates studies that have revealed that information credibility influences the adoption of COVID-19 information (Mensah et al., 2022).

Additionally, the direct impact of COVID-19 information usefulness on the adoption of COVID-19 pandemic information on social media systems demonstrates that only COVID-19 information that is useful to users to understand the preventive and control measures and to protect themselves and loved ones from the deadly virus will be adopted. Therefore, COVID-19 information management authorities should be developed to disseminate relevant COVID-19 information (such as where to get vaccinated, infested citizens/regions, preventive and control measures/policies, COVID-19 testing, etc.) in a more meaningful and effective manner to fight the pandemics and saving precious lives (i.e., information saves lives). This result is also supported by the previous research that information usefulness influences the adoption of COVID-19 information (Han et al., 2021; Mensah et al., 2022).

The study’s validation of the positive moderating influence of perceived government information transparency on the impact of information quality, information credibility, and information usefulness on the adoption of COVID-19 information on social media systems is a strong testament to the critical role government information transparency regarding the management of COVID-19 pandemic which can drive people’s adoption behavior of such information. Consequently, transparency in COVID-19 information dissemination can enhance the predictive power of both information quality and information usefulness on the citizens’ adoption of COVID-19 information on social media. COVID-19 information communication should promote information that is in the public interest, factual, transparent, and devoid of any political communication. This is important since any form of political polarization and fragmentation of COVID-19 information as exists in many countries (especially in Ghana and other African countries) can cause people to refuse to adopt official government information if they consider it to be politicized. Also, transparency in COVID-19 communications, management, and obligation on the part of the government could be fundamental for citizens to hold governments accountable after the pandemic ends. For COVID-19 information to be effective and ensure greater public trust in government it should be based on standards of transparency, integrity, and accountability. It is important to stress that transparency in government COVID-19 information dissemination can be instrumental to support COVID-19 policy measures and combat waves of disinformation regarding the pandemic. The findings on the positive moderating influence of government information transparency on the impact of information quality, credibility, and usefulness on the adoption of COVID-19 pandemic information could not be compared with any past or current literature since it is a novel addition to the COVID-19 information adoption literature.

### 6.2. Theoretical implications

The integration of perceived government information transparency (PGIT) into the Information Adoption Model (IAM) as a moderator construct contributes to the literature and theoretical model to understand public health information adoption during a global crisis like the COVID-19 pandemic. The validated model has shown that government information transparency positively moderates the major constructs of the IAM such as information quality, information credibility, and information usefulness on citizens’ adoption of COVID-19 information on social media systems. These findings provide new perspectives for researchers to continue to validate, modify and extend our model in light of the Information Adoption Model (IAM) to explore the adoption of COVID-19 pandemic information on social media systems and in particular any other public health emergencies that may occur in the future.

## 7. Conclusion

This paper examines the moderated influence of perceived government information transparency on the adoption of COVID-19 pandemic information on social media systems based on the information adoption model’s principles. Social media systems came in handy for the government and its public health officials to use to manage and communicate information about the COVID-19 pandemic to the general public. Thus, government information transparency regarding the management and information disclosure about the COVID-19 pandemic is crucial to enhance the maximum cooperation of citizens toward the adoption of COVID-19 policies and preventive measures. Consequently, this study based on data generated from the Ghana context has demonstrated that information quality, information credibility, and information usefulness have a direct significant impact on the adoption of COVID-19 information on social media. Additionally, perceived government information transparency was found to moderate positively the influence of information quality, information credibility, and information usefulness, respectively, on the citizens’ adoption of COVID-19 pandemic information. These findings highlight the relevance of government information transparency concerning the pandemic on the management of the COVID-19 pandemic and its subsequent impact on shaping people’s behavior toward government as well as pandemic information. Government COVID-19 information transparency can be accomplished by the provision of detailed information on all aspects of the COVID-19 pandemic such as epidemiological information (number of people infected, deaths, and tests), information on decisions made by public administrators (hospital bed availability, occupancy rate, and contact tracing, etc.) and financial budgetary information (budget allocated, donations, emergency acquisitions, transfer of assets, etc.).

## 8. Limitations and future research

The issues surrounding government transparency in COVID-19 information management vary across different country settings and thus although the models and processes used in our study may be applied in the context of other developing countries, the results and conclusions may not necessarily conform to our findings and conclusions. Thus, caution should be exercised in the interpretation and generalization of the findings as study uses convenient sampling technique. Furthermore, not all the factors driving the adoption of COVID-19 pandemic information on social media systems were tested in this study and thus future research is warranted to integrate other factors into the IAM model. Future research could combine the IAM and Unified Theory of Acceptance and Use of Technology (UTAUT) constructs such as performance expectancy, effort expectancy, social influence, and facilitation conditions along with trust in government to understand COVID-19 pandemic information adoption. Additionally, individual personal characteristics such as gender, age, education, etc. can influence the adoption of the COVID-19 pandemic on social systems which are not statistically significant in ours study. The low *R*^2^ indicates that there may be some other relevant predictors that can enhance model fit. Therefore, the relatively low R^2^ reported in this study could motivate other researchers to continue to validate our model to obtain an improved R^2^ results.

## Data availability statement

The raw data supporting the conclusions of this article will be made available by the authors, without undue reservation.

## Ethics statement

Ethical review and approval of human participants were not required for this study in accordance with local legislation and institutional requirements. However, written informed consent from the participants was obtained in accordance with the national legislation and institutional requirements.

## Author contributions

IM and DM: conceptualization, methodology, data collection, and draft write-up. IM, JL, and DM: project administration. MK: data analysis and interpretation. MK and L-WL: proofreading/editing. JL and NZ: project funding. All authors contributed to the article and approved the submitted version.

## Funding

This work was supported by the National Nature Science Foundation of China (grant number 71902034), National Science Foundation Cultivation Program of Fujian Jiangxia University (JXS2021007), Educational and Scientific Research Project for Young and Middle-aged Teachers of Fujian Province (JAT210365) and Natural Science Foundation of Fujian Province (2022J01955).

## Conflict of interest

The authors declare that the research was conducted in the absence of any commercial or financial relationships that could be construed as a potential conflict of interest.

## Publisher’s note

All claims expressed in this article are solely those of the authors and do not necessarily represent those of their affiliated organizations, or those of the publisher, the editors and the reviewers. Any product that may be evaluated in this article, or claim that may be made by its manufacturer, is not guaranteed or endorsed by the publisher.
